# Stochasticity Shapes Microbial Communities in High‐Altitude Lakes, Whereas Species Selection and Homogenization Dispersal Are More Important in Lowland Lakes: Case of Benthic Diatoms in Alpine Lakes

**DOI:** 10.1002/ece3.71977

**Published:** 2025-08-27

**Authors:** F. Rimet, C. Lemonnier, B. Alric

**Affiliations:** ^1^ Université Savoie‐Mont Blanc, INRAE UMR CARRTEL Thonon‐les‐Bains France; ^2^ Pôle R&D ECLA INRAE OFB Thonon‐les‐Bains France

**Keywords:** dispersion, homogenization, lake, metabarcoding, microalgae, selection

## Abstract

Lakes are fundamental ecosystems for human well‐being, and understanding the ecological processes shaping their biological communities is of prime importance. Among microalgae, diatoms are often dominating the biomass of their benthic habitats, especially in European alpine lakes. We hypothesized that, because of their contrasted hydro‐morphometric characteristics, high‐altitude and lowland lake communities may have contrasted diversities and may be shaped differently by ecological processes. We sampled benthic diatoms in 46 high‐altitude and 12 lowland lakes in the framework of several projects between years 2013 and 2019. Each lake was sampled in several places along their shoreline depending on lake size, for a total of 514 samples. Samples were analyzed with DNA metabarcoding and specific diatom primers. We used a naïve taxonomic approach based on amplicon sequence variant and a method based on null modelling and phylogenetic indices, together with variance partitioning and co‐occurrence networks. Lowland lakes had higher alpha diversities, and the irdiatom ecological networks were more connected than high‐altitude lakes. These distinct structural patterns between lake types were explained by different contributions of ecological processes. Although dispersal was similarly restricted in both lake types, diatom communities in high‐altitude lakes were mainly governed by stochastic processes, which was not the case in lowlands. This pattern can be explained by the extreme conditions at high altitudes, which create major bottlenecks in diatom communities and thus favour stochasticity. Conversely, homogenizing dispersion was greater in lowlands, confirming the hypothesis of greater connectivity via anthropogenic and biotic vectors in lowland lakes. These findings suggest that high‐altitude lakes could be less resilient to environmental changes due to lower diversity and network connectivity compared with lowlands. Finally, we advocate the combined use of diversity metrics, statistical analyses to assess ecological processes, and inference of networks because they provide complementary information to understand lakes vulnerabilities.

## Graphical abstract

Lakes microalgal communities are shaped by differing ecological processes depending on their altitude. Based on benthic samples of biofilms analyzed with metabarcoding in several European countries around the Alps, we showed that high‐altitude lake communities are driven by stochastic processes, whereas in lowlands they are driven by homogenizing dispersal. These differences may have an impact on lakes resilience, especially in high‐altitude, where local diversity is smaller and ecological networks are less connected.

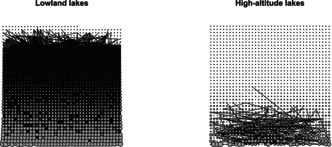

## 1 Introduction

Lakes are fundamental ecosystems for human well‐being because they provide a number of essential socio‐economic services (Millennium Ecosystem Assessment 2005; Dudgeon et al. 2006). However, they face significant threats from intensified human activities (Mammides 2020). Understanding the ecological processes driving lakes biodiversity is essential for the sustainable management of these ecosystems. Benthic diatoms, a highly diverse group of microalgae (~100,000 species globally; Mann and Vanormelingen 2013), are key contributors in the littoral zone, respond rapidly to environmental changes (Rimet 2012), and are often used as lake ecological indicators (Marchetto et al. 2013).

Selection, dispersal, speciation, and ecological drift are the four fundamental ecological processes regulating the diversity of communities (Vellend 2010). The relative importance of these processes in shaping lake diatom communities has been investigated for the past decades. Traditionally, research on microalgal communities, including diatoms, and the factors driving changes in their composition focused mainly on local environmental changes (Butcher 1947; Fjerdingstad 1950). The ecological process of species selection when referring to environmental filtering (Vellend 2010) has long been described as the most important process in explaining variability in diatom communities, and a large body of research has described this process in freshwater ecosystems (Rimet 2012; Huber et al. 2020). In particular, it has been shown that under the species selection process, parameters such as nutrients and organic matter concentration in water (Griffiths et al. 2002) as well as grazing (de Faria et al. 2017) influenced diatom community composition. These results led to the development of monitoring tools for assessing river (e.g., Prygiel and Coste 1996; Stevenson and Bahls 2002) and lake pollution (e.g., Hoffman 1994; Marchetto et al. 2009).

Spatial factors and dispersal limitation gained attention in diatom research later than species selection, in the 21st century, with the debate on the Baas‐Becking ubiquity hypothesis (everything is everywhere) applied to diatoms (Finlay et al. 2002). Observation of endemic diatom taxa in ancient lakes (Kulikovskiy and Lange‐Bertalot 2012; Levkov et al. 2007) and lakes located in isolated regions such as Tasmania and New Zealand (Vyverman et al. 2007) challenged this hypothesis. Recent metabarcoding studies revealed limited dispersal capacity at the subspecies level of diatoms, with, for example, high‐altitude lakes separated by 3000 km sharing only 13% of subspecies (Rimet, Pinseel, et al. 2023). While diatoms are now recognized to have dispersal limits and geographic patterns (Pinseel et al. 2024), some species, like *Didymosphaenia geminata* (Blanco and Ector 2009) and tropical invasive diatoms (Coste and Ector 2000), exhibit widespread invasions in rivers. Diatom species invasions in lakes are less known but were revealed using a paleolimnological approach (Alverson et al. 2021). The mass‐effect process, focused on the dispersal of individuals from source to sink environments, strongly influences lake communities through cells deriving from tributaries into the lake (Benito et al. 2018; Rimet, Canino, et al. 2023).

Speciation (or diversification) is another central process in shaping biodiversity (Vellend 2010), yet often overlooked in community ecology despite its importance (Zhou and Ning 2017). Addressing this gap is crucial for understanding protist and diatom ecology (Pinseel et al. 2024). In ancient lakes, the importance of speciation is evident through “species flocks,” which are rapid diversification events of particular taxa (Kociolek et al. 2017). For instance, in Lake Ochrid, molecular and fossil evidence shows a rapid diversification of the genus *Aneumastus*, resulting in many endemic species and a particular taxonomic composition of its communities (Stelbrink et al. 2018).

Finally, ecological drift involves stochastic changes in community composition over time due to random processes like birth, death, and reproduction, especially in small or finite populations (Vellend 2010). Although microbial communities are typically large, the many rare taxa within them are vulnerable to ecological drift (Zhou and Ning 2017). Stochasticity has been increasingly studied in diatom communities over the past 15 years, with special attention in alpine rivers affected by stochastic hydrological events (Centis et al. 2010) and during species succession in rivers (Passy and Larson 2011). Its importance grows with geographical scale in dispersal‐related factors (Soininen 2023) and is dominant in alpine mountain lakes for littoral and plankton diatom communities (Ossyssek et al. 2023).

Lakes of the Alpine region provide important ecosystem services to neighboring populations (Baulaz et al. 2021). Due to the typological differences (some being large and low‐lying, others small and high‐lying) of lakes in this region, their biological communities can be structured by different ecological processes. In the current context of global change, understanding which ecological processes control the biological communities of these lakes will enable us to anticipate their evolution. Our aim is to evaluate how ecological processes influence diatom communities in contrasting lake types (high‐altitude vs. lowland lakes) within the same climatic (Alpine) and geographical (Western Europe) zone. Given their contrasted hydro‐morphometric characteristics (depth, altitude, residence time, lake surface, and catchment area), we hypothesize that these lakes will exhibit distinct alpha, beta, and gamma diversities, with varying ecological processes shaping these patterns. Our working hypotheses are as follows:

1. High‐altitude lakes are pioneer ecosystems with harsher climatic and physical conditions (e.g., freezing, drying) compared to lowlands. Therefore, our 1st hypothesis was that stochastic processes (e.g., ecological drift) dominate in high‐altitude lakes, while deterministic processes (selection, dispersal) are more significant in lowlands.
2. High‐altitude lake communities exhibit high endemism and limited dispersal capacities due to geographical barriers (Rimet, Pinseel, et al. 2023). In contrast, lowland lakes should be less affected by these barriers due to anthropogenic and biotic vectors (e.g., ballast water, animal movements) that facilitate diatom transfer, even between lakes not hydrographically connected. Consequently, our 2nd hypothesis was that lowland lakes share more species, have lower beta‐diversity, and show greater community similarity than high‐altitude lakes.
3. Diatom communities in lakes are influenced by mass‐effect from rivers arriving in them (Rimet, Canino, et al. 2023). Lakes with large catchment areas attract more dispersers than smaller ecosystems, enhancing diversity and richness (Gilpin and Diamond 1976). Since high‐altitude lakes usually have smaller catchment areas than lowland lakes, our 3rd hypothesis was that lowland lakes host a higher diversity (when considering each lake's diversity separately) and that there was a correlation between lake diversity and the lake's surface and the lake's watershed surface.
4. If these hypotheses of stronger geographical barriers and stochasticity in high‐altitude lakes are confirmed, our 4th hypothesis was that ecological networks were more complex in lowlands with a higher number of co‐occurring taxa, as well as stronger connectivity and connectance. Using ecological network metrics will provide complementary insights to diversity metrics, in particular concerning the stability and resilience of ecosystems (Landi et al. 2018).

To test these hypotheses, while avoiding climate‐related confounding factors, we sampled 56 high‐altitude and lowland lakes located in the same climatic zone, the European alpine region. Benthic diatom communities, sampled in the littoral zone, were analyzed by DNA metabarcoding targeting a 263 base‐pairs fragment of a chloroplast gene (rbcL). This fragment is known to be resolutive at the subspecific level (Kermarrec et al. 2013). In addition, the reference barcode library ‐Diat.barcode‐ dedicated to this marker gene and curated by a network of experts for 14 years is available (Rimet, Gusev, et al. 2019). Since species boundaries can be ambiguous, we used a naïve taxonomic approach based on the sequence variants (ASV, amplicon sequence variants). We compared communities' similarities and alpha and beta diversities between lake types. Using a reference phylogeny where environmental sequences were relocated, we quantified each ecological process for each lake type following the approach proposed by Stegen et al. (2013). Additionally, variance partitioning was used to assess environmental and geographical effects on community variability, and co‐occurrence network metrics were compared across lake types.

## 2 Materials and Methods

### 2.1 Study Area and Samplings

Two types of lakes were monitored, high‐altitude and lowland lakes, where benthic diatoms were sampled according to the European guidelines for diatom metabarcoding sampling (CEN 2025). For each sample, five stones were collected at a depth of 40–50 cm in an area of 20 m^2^, the upper surface of which was scraped with a clean toothbrush (minimal surface of 100 cm^2^) to recover the biofilms in sterile plastic tubes. We then fixed the biofilms with a minimal final concentration of 70% ethanol before any further treatment.

**FIGURE 1:**
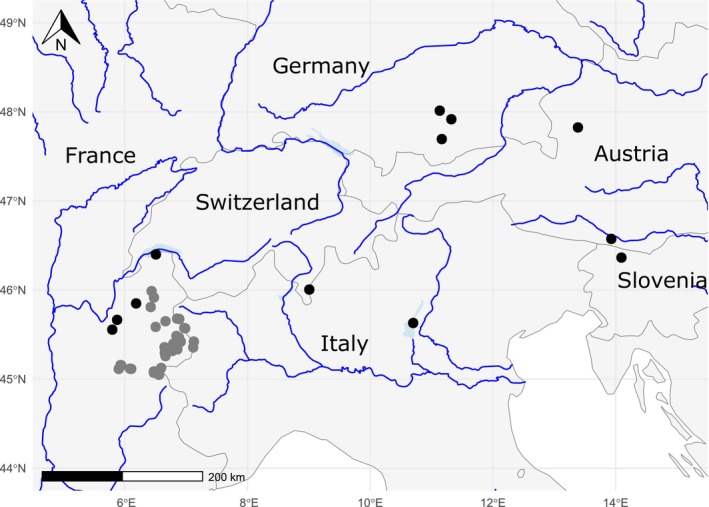
Location of sampling sites in the Alps mountains. A total of 12 lowland lakes (black dot) and 46 high‐altitude lakes (gray dots) were sampled. Blue lines: rivers; light blue: large lake in; gray lines: countries boundaries.

Forty‐six high‐altitude lakes were sampled in the northern (23 lakes) and mid‐southern (23 lakes) French Alps, above 1500 m a.s.l. in summer 2013 (aquatic ecosystems above 800 m asl are considered high‐altitude in system A of the European Water Framework Directive). For each lake, we collected three samples, except eight lakes where only two samples were taken. The position along the shore of the sampling stations was chosen so that the distance between them was maximized. Twelve lowland lakes (below 800 m asl) were sampled in the alpine region of several European countries (Austria, France, Germany, Italy, Slovenia, Switzerland), with altitudes ranging from 65 to 649 m during the summer and early autumn period from 2017 to 2019. For each lake, from 3 to 152 sampling stations were taken (average of 32). These sampling stations were chosen along the shoreline of the lakes in order to capture a maximum variety of situations, the number of sampling sites being adapted to the lake's surface (Rimet, Canino, et al. 2023). The location of the lakes is given in Figure 1; a total of 125 samples were collected in high‐altitude lakes and 389 in lowland lakes. Table 1 gives the geographical position of the sampling sites as well as the dates of sampling for each lake (for complete metadata with physico‐chemical parameters, as well as lake characteristics—altitude, lake depth, surface, watershed surface, and residence time—see Supporting Information S1).

**TABLE 1:** Lakes sampled, with their sampling sites position, sampling dates and elevation.

Lake name	Station	Country	Latitude (GPS)	Longitude (GPS)	Sampling date	Elevation (m asl)
Annecy	Annecy_1	FR	45.86094	6.14480	17/07/2019	447
Annecy	Annecy_2	FR	45.84714	6.15560	16/07/2019	447
Annecy	Annecy_3	FR	45.84474	6.17406	17/07/2019	447
Annecy	Annecy_4	FR	45.83944	6.17777	16/07/2019	447
Annecy	Annecy_5	FR	45.83398	6.18392	16/07/2019	447
Annecy	Annecy_6	FR	45.83078	6.18958	16/07/2019	447
Annecy	Annecy_7	FR	45.82855	6.20013	16/07/2019	447
Annecy	Annecy_9	FR	45.82148	6.20773	16/07/2019	447
Annecy	Annecy_10	FR	45.81443	6.21031	16/07/2019	447
Annecy	Annecy_11	FR	45.80522	6.21211	16/07/2019	447
Annecy	Annecy_12	FR	45.79317	6.21585	16/07/2019	447
Annecy	Annecy_13	FR	45.79478	6.22152	16/07/2019	447
Annecy	Annecy_14	FR	45.79711	6.22658	16/07/2019	447
Annecy	Annecy_15	FR	45.79996	6.22906	16/07/2019	447
Annecy	Annecy_16	FR	45.80543	6.23155	16/07/2019	447
Annecy	Annecy_17	FR	45.81149	6.23049	16/07/2019	447
Annecy	Annecy_18	FR	45.81597	6.22860	16/07/2019	447
Annecy	Annecy_19	FR	45.82298	6.22143	16/07/2019	447
Annecy	Annecy_20	FR	45.82732	6.21808	16/07/2019	447
Annecy	Annecy_21	FR	45.83656	6.21500	16/07/2019	447
Annecy	Annecy_22	FR	45.84186	6.20820	16/07/2019	447
Annecy	Annecy_23	FR	45.84367	6.18871	16/07/2019	447
Annecy	Annecy_24	FR	45.85323	6.18737	17/07/2019	447
Annecy	Annecy_25	FR	45.85943	6.18498	17/07/2019	447
Annecy	Annecy_26	FR	45.86252	6.18555	17/07/2019	447
Annecy	Annecy_27	FR	45.87101	6.18060	17/07/2019	447
Annecy	Annecy_28	FR	45.88066	6.17448	17/07/2019	447
Annecy	Annecy_29	FR	45.89134	6.16685	17/07/2019	447
Annecy	Annecy_30	FR	45.89874	6.16200	17/07/2019	447
Annecy	Annecy_31	FR	45.90289	6.15991	17/07/2019	447
Annecy	Annecy_32	FR	45.90428	6.15174	17/07/2019	447
Annecy	Annecy_33	FR	45.90206	6.14475	17/07/2019	447
Annecy	Annecy_34	FR	45.90134	6.13334	17/07/2019	447
Annecy	Annecy_35	FR	45.89740	6.13376	17/07/2019	447
Annecy	Annecy_36	FR	45.89235	6.13850	17/07/2019	447
Annecy	Annecy_37	FR	45.89024	6.14350	17/07/2019	447
Annecy	Annecy_38	FR	45.87779	6.14328	17/07/2019	447
Annecy	Annecy_39	FR	45.87150	6.14419	17/07/2019	447
Léman (Lake Geneva)	SYN1	FR/CH	46.20904	6.16238	27/06/2017	375
Léman (Lake Geneva)	SYN10	FR/CH	46.30400	6.18173	27/06/2017	375
Léman (Lake Geneva)	SYN100	FR/CH	46.39892	6.76829	14/06/2017	375
Léman (Lake Geneva)	SYN101	FR/CH	46.40553	6.73323	14/06/2017	375
Léman (Lake Geneva)	SYN102	FR/CH	46.40776	6.71082	14/06/2017	375
Léman (Lake Geneva)	SYN103	FR/CH	46.40783	6.68956	14/06/2017	375
Léman (Lake Geneva)	SYN104	FR/CH	46.40502	6.66787	14/06/2017	375
Léman (Lake Geneva)	SYN105	FR/CH	46.40562	6.64282	14/06/2017	375
Léman (Lake Geneva)	SYN106	FR/CH	46.40442	6.62526	14/06/2017	375
Léman (Lake Geneva)	SYN107	FR/CH	46.40307	6.60698	14/06/2017	375
Léman (Lake Geneva)	SYN108	FR/CH	46.40302	6.60720	14/06/2017	375
Léman (Lake Geneva)	SYN109	FR/CH	46.39977	6.57290	14/06/2017	375
Léman (Lake Geneva)	SYN11	FR/CH	46.31891	6.19622	27/06/2017	375
Léman (Lake Geneva)	SYN110	FR/CH	46.39546	6.54316	14/06/2017	375
Léman (Lake Geneva)	SYN111	FR/CH	46.40203	6.52576	14/06/2017	375
Léman (Lake Geneva)	SYN112	FR/CH	46.40361	6.52225	15/06/2017	375
Léman (Lake Geneva)	SYN113	FR/CH	46.40392	6.51998	15/06/2017	375
Léman (Lake Geneva)	SYN114	FR/CH	46.40427	6.51875	15/06/2017	375
Léman (Lake Geneva)	SYN115	FR/CH	46.40476	6.51651	15/06/2017	375
Léman (Lake Geneva)	SYN116	FR/CH	46.40380	6.50802	15/06/2017	375
Léman (Lake Geneva)	SYN117	FR/CH	46.39529	6.48699	15/06/2017	375
Léman (Lake Geneva)	SYN118	FR/CH	46.37880	6.47949	15/06/2017	375
Léman (Lake Geneva)	SYN119	FR/CH	46.37884	6.47943	15/06/2017	375
Léman (Lake Geneva)	SYN12	FR/CH	46.33671	6.20551	27/06/2017	375
Léman (Lake Geneva)	SYN120	FR/CH	46.37454	6.47406	15/06/2017	375
Léman (Lake Geneva)	SYN121	FR/CH	46.36803	6.45378	15/06/2017	375
Léman (Lake Geneva)	SYN122	FR/CH	46.36423	6.44355	15/06/2017	375
Léman (Lake Geneva)	SYN123	FR/CH	46.36410	6.44302	15/06/2017	375
Léman (Lake Geneva)	SYN124	FR/CH	46.36389	6.44243	15/06/2017	375
Léman (Lake Geneva)	SYN125	FR/CH	46.35923	6.41189	15/06/2017	375
Léman (Lake Geneva)	SYN126	FR/CH	46.35021	6.39925	15/06/2017	375
Léman (Lake Geneva)	SYN127	FR/CH	46.34985	6.39885	15/06/2017	375
Léman (Lake Geneva)	SYN128	FR/CH	46.34864	6.39874	15/06/2017	375
Léman (Lake Geneva)	SYN129	FR/CH	46.34425	6.39404	15/06/2017	375
Léman (Lake Geneva)	SYN13	FR/CH	46.34973	6.20982	27/06/2017	375
Léman (Lake Geneva)	SYN130	FR/CH	46.34331	6.37656	15/06/2017	375
Léman (Lake Geneva)	SYN131	FR/CH	46.34314	6.37570	15/06/2017	375
Léman (Lake Geneva)	SYN132	FR/CH	46.34352	6.37450	15/06/2017	375
Léman (Lake Geneva)	SYN133	FR/CH	46.35355	6.35709	15/06/2017	375
Léman (Lake Geneva)	SYN134	FR/CH	46.36232	6.35038	15/06/2017	375
Léman (Lake Geneva)	SYN135	FR/CH	46.36973	6.34016	15/06/2017	375
Léman (Lake Geneva)	SYN136	FR/CH	46.36909	6.31815	16/06/2017	375
Léman (Lake Geneva)	SYN137	FR/CH	46.36274	6.29873	16/06/2017	375
Léman (Lake Geneva)	SYN138	FR/CH	46.35352	6.28315	16/06/2017	375
Léman (Lake Geneva)	SYN139	FR/CH	46.34237	6.27469	16/06/2017	375
Léman (Lake Geneva)	SYN14	FR/CH	46.36075	6.21972	27/06/2017	375
Léman (Lake Geneva)	SYN140	FR/CH	46.32670	6.25942	16/06/2017	375
Léman (Lake Geneva)	SYN141	FR/CH	46.31553	6.25552	16/06/2017	375
Léman (Lake Geneva)	SYN142	FR/CH	46.30458	6.24208	16/06/2017	375
Léman (Lake Geneva)	SYN143	FR/CH	46.30429	6.24172	16/06/2017	375
Léman (Lake Geneva)	SYN144	FR/CH	46.30373	6.24068	16/06/2017	375
Léman (Lake Geneva)	SYN145	FR/CH	46.29086	6.23679	16/06/2017	375
Léman (Lake Geneva)	SYN146	FR/CH	46.28014	6.22333	16/06/2017	375
Léman (Lake Geneva)	SYN147	FR/CH	46.26986	6.21415	16/06/2017	375
Léman (Lake Geneva)	SYN148	FR/CH	46.26431	6.19999	16/06/2017	375
Léman (Lake Geneva)	SYN149	FR/CH	46.25344	6.19335	16/06/2017	375
Léman (Lake Geneva)	SYN15	FR/CH	46.37001	6.23141	27/06/2017	375
Léman (Lake Geneva)	SYN150	FR/CH	46.23676	6.19235	16/06/2017	375
Léman (Lake Geneva)	SYN151	FR/CH	46.22781	6.18543	16/06/2017	375
Léman (Lake Geneva)	SYN152	FR/CH	46.21766	6.17696	16/06/2017	375
Léman (Lake Geneva)	SYN153	FR/CH	46.20894	6.16259	27/06/2017	375
Léman (Lake Geneva)	SYN16	FR/CH	46.38368	6.24671	27/06/2017	375
Léman (Lake Geneva)	SYN17	FR/CH	46.38753	6.24863	27/06/2017	375
Léman (Lake Geneva)	SYN18	FR/CH	46.39102	6.25405	27/06/2017	375
Léman (Lake Geneva)	SYN19	FR/CH	46.39291	6.26256	27/06/2017	375
Léman (Lake Geneva)	SYN2	FR/CH	46.21017	6.15694	27/06/2017	375
Léman (Lake Geneva)	SYN20	FR/CH	46.39125	6.26712	26/06/2017	375
Léman (Lake Geneva)	SYN21	FR/CH	46.39025	6.27420	26/06/2017	375
Léman (Lake Geneva)	SYN22	FR/CH	46.40700	6.28306	26/06/2017	375
Léman (Lake Geneva)	SYN23	FR/CH	46.42246	6.29075	26/06/2017	375
Léman (Lake Geneva)	SYN24	FR/CH	46.42440	6.29701	26/06/2017	375
Léman (Lake Geneva)	SYN25	FR/CH	46.42553	6.29690	26/06/2017	375
Léman (Lake Geneva)	SYN26	FR/CH	46.43260	6.30967	26/06/2017	375
Léman (Lake Geneva)	SYN27	FR/CH	46.44326	6.31924	26/06/2017	375
Léman (Lake Geneva)	SYN28	FR/CH	46.46146	6.34772	26/06/2017	375
Léman (Lake Geneva)	SYN29	FR/CH	46.46415	6.36893	26/06/2017	375
Léman (Lake Geneva)	SYN3	FR/CH	46.22596	6.14949	27/06/2017	375
Léman (Lake Geneva)	SYN30	FR/CH	46.46390	6.38565	26/06/2017	375
Léman (Lake Geneva)	SYN31	FR/CH	46.46161	6.38928	26/06/2017	375
Léman (Lake Geneva)	SYN32	FR/CH	46.45978	6.39139	26/06/2017	375
Léman (Lake Geneva)	SYN33	FR/CH	46.45910	6.39756	26/06/2017	375
Léman (Lake Geneva)	SYN34	FR/CH	46.45995	6.40530	26/06/2017	375
Léman (Lake Geneva)	SYN35	FR/CH	46.46712	6.41748	26/06/2017	375
Léman (Lake Geneva)	SYN36	FR/CH	46.47127	6.43872	26/06/2017	375
Léman (Lake Geneva)	SYN37	FR/CH	46.47551	6.45005	26/06/2017	375
Léman (Lake Geneva)	SYN38	FR/CH	46.49107	6.47923	26/06/2017	375
Léman (Lake Geneva)	SYN39	FR/CH	46.50358	6.49333	26/06/2017	375
Léman (Lake Geneva)	SYN4	FR/CH	46.24355	6.15302	27/06/2017	375
Léman (Lake Geneva)	SYN40	FR/CH	46.50403	6.49456	26/06/2017	375
Léman (Lake Geneva)	SYN41	FR/CH	46.50430	6.49560	26/06/2017	375
Léman (Lake Geneva)	SYN42	FR/CH	46.51245	6.50157	26/06/2017	375
Léman (Lake Geneva)	SYN43	FR/CH	46.51512	6.51330	22/06/2017	375
Léman (Lake Geneva)	SYN44	FR/CH	46.51229	6.52844	22/06/2017	375
Léman (Lake Geneva)	SYN45	FR/CH	46.50741	6.53873	22/06/2017	375
Léman (Lake Geneva)	SYN46	FR/CH	46.50724	6.53919	22/06/2017	375
Léman (Lake Geneva)	SYN47	FR/CH	46.50727	6.53981	22/06/2017	375
Léman (Lake Geneva)	SYN48	FR/CH	46.50742	6.54003	22/06/2017	375
Léman (Lake Geneva)	SYN49	FR/CH	46.50761	6.55827	22/06/2017	375
Léman (Lake Geneva)	SYN5	FR/CH	46.26238	6.16629	27/06/2017	375
Léman (Lake Geneva)	SYN50	FR/CH	46.51368	6.57259	22/06/2017	375
Léman (Lake Geneva)	SYN51	FR/CH	46.51766	6.58531	22/06/2017	375
Léman (Lake Geneva)	SYN52	FR/CH	46.51727	6.58684	22/06/2017	375
Léman (Lake Geneva)	SYN53	FR/CH	46.51743	6.58803	22/06/2017	375
Léman (Lake Geneva)	SYN54	FR/CH	46.51487	6.59951	22/06/2017	375
Léman (Lake Geneva)	SYN55	FR/CH	46.51145	6.60987	22/06/2017	375
Léman (Lake Geneva)	SYN56	FR/CH	46.50573	6.62451	22/06/2017	375
Léman (Lake Geneva)	SYN57	FR/CH	46.50574	6.62461	22/06/2017	375
Léman (Lake Geneva)	SYN58	FR/CH	46.50688	6.63413	22/06/2017	375
Léman (Lake Geneva)	SYN59	FR/CH	46.50573	6.65215	22/06/2017	375
Léman (Lake Geneva)	SYN6	FR/CH	46.27388	6.17142	27/06/2017	375
Léman (Lake Geneva)	SYN60	FR/CH	46.50398	6.67302	22/06/2017	375
Léman (Lake Geneva)	SYN61	FR/CH	46.50048	6.69035	22/06/2017	375
Léman (Lake Geneva)	SYN62	FR/CH	46.49808	6.70010	22/06/2017	375
Léman (Lake Geneva)	SYN63	FR/CH	46.48850	6.71840	22/06/2017	375
Léman (Lake Geneva)	SYN64	FR/CH	46.48920	6.73753	22/06/2017	375
Léman (Lake Geneva)	SYN65	FR/CH	46.48633	6.75167	21/06/2017	375
Léman (Lake Geneva)	SYN66	FR/CH	46.47880	6.76451	21/06/2017	375
Léman (Lake Geneva)	SYN67	FR/CH	46.47401	6.78514	21/06/2017	375
Léman (Lake Geneva)	SYN68	FR/CH	46.47101	6.80548	21/06/2017	375
Léman (Lake Geneva)	SYN69	FR/CH	46.46837	6.82820	21/06/2017	375
Léman (Lake Geneva)	SYN7	FR/CH	46.27481	6.17092	27/06/2017	375
Léman (Lake Geneva)	SYN70	FR/CH	46.46343	6.83490	21/06/2017	375
Léman (Lake Geneva)	SYN71	FR/CH	46.46079	6.83489	21/06/2017	375
Léman (Lake Geneva)	SYN72	FR/CH	46.45517	6.85289	21/06/2017	375
Léman (Lake Geneva)	SYN73	FR/CH	46.44807	6.87065	21/06/2017	375
Léman (Lake Geneva)	SYN74	FR/CH	46.43974	6.88882	21/06/2017	375
Léman (Lake Geneva)	SYN75	FR/CH	46.43955	6.90310	21/06/2017	375
Léman (Lake Geneva)	SYN76	FR/CH	46.43067	6.90892	21/06/2017	375
Léman (Lake Geneva)	SYN77	FR/CH	46.42297	6.92359	21/06/2017	375
Léman (Lake Geneva)	SYN78	FR/CH	46.41317	6.92906	21/06/2017	375
Léman (Lake Geneva)	SYN79	FR/CH	46.40233	6.92507	21/06/2017	375
Léman (Lake Geneva)	SYN8	FR/CH	46.27518	6.17083	27/06/2017	375
Léman (Lake Geneva)	SYN80	FR/CH	46.39725	6.91646	19/06/2017	375
Léman (Lake Geneva)	SYN81	FR/CH	46.39861	6.90728	19/06/2017	375
Léman (Lake Geneva)	SYN82	FR/CH	46.39606	6.88567	19/06/2017	375
Léman (Lake Geneva)	SYN83	FR/CH	46.39382	6.87077	21/06/2017	375
Léman (Lake Geneva)	SYN84	FR/CH	46.39470	6.86063	21/06/2017	375
Léman (Lake Geneva)	SYN85	FR/CH	46.39539	6.86017	21/06/2017	375
Léman (Lake Geneva)	SYN86	FR/CH	46.39518	6.85988	19/06/2017	375
Léman (Lake Geneva)	SYN88	FR/CH	46.39428	6.85856	19/06/2017	375
Léman (Lake Geneva)	SYN89	FR/CH	46.39455	6.85813	19/06/2017	375
Léman (Lake Geneva)	SYN9	FR/CH	46.28995	6.17067	27/06/2017	375
Léman (Lake Geneva)	SYN90	FR/CH	46.39458	6.85772	19/06/2017	375
Léman (Lake Geneva)	SYN91	FR/CH	46.39380	6.85717	19/06/2017	375
Léman (Lake Geneva)	SYN92	FR/CH	46.38987	6.85888	19/06/2017	375
Léman (Lake Geneva)	SYN93	FR/CH	46.38723	6.85098	19/06/2017	375
Léman (Lake Geneva)	SYN94	FR/CH	46.38674	6.83632	19/06/2017	375
Léman (Lake Geneva)	SYN95	FR/CH	46.38906	6.81755	19/06/2017	375
Léman (Lake Geneva)	SYN96	FR/CH	46.39383	6.80574	19/06/2017	375
Léman (Lake Geneva)	SYN97	FR/CH	46.39419	6.80539	19/06/2017	375
Léman (Lake Geneva)	SYN98	FR/CH	46.39431	6.80436	19/06/2017	375
Léman (Lake Geneva)	SYN99	FR/CH	46.39426	6.78619	19/06/2017	375
Bled	Sample10	SI	46.36288	14.08240	12/08/2019	475
Bled	Sample1	SI	46.36191	14.08485	12/08/2019	475
Bled	Sample2	SI	46.35919	14.08549	12/08/2019	475
Bled	Sample3	SI	46.36294	14.09024	12/08/2019	475
Bled	Sample4	SI	46.36269	14.08961	12/08/2019	475
Bled	Sample5	SI	46.36254	14.09943	12/08/2019	475
Bled	Sample6	SI	46.36373	14.10294	12/08/2019	475
Bled	Sample7	SI	46.36503	14.10622	12/08/2019	475
Bled	Sample8	SI	46.36878	14.10787	12/08/2019	475
Bled	Sample9	SI	46.36633	14.08609	12/08/2019	475
Ammersee	A1	DE	47.98609	11.16236	01/07/2019	533
Ammersee	A11	DE	48.00937	11.09907	09/07/2019	533
Ammersee	A13	DE	48.04163	11.09776	09/07/2019	533
Ammersee	A14	DE	48.06256	11.10509	09/07/2019	533
Ammersee	A15	DE	48.07385	11.12242	09/07/2019	533
Ammersee	A16	DE	48.06502	11.13839	09/07/2019	533
Ammersee	A17	DE	48.03729	11.13113	09/07/2019	533
Ammersee	A18	DE	48.00858	11.14056	10/07/2019	533
Ammersee	A19	DE	48.00217	11.15805	09/07/2019	533
Ammersee	A2	DE	47.96773	11.14929	09/07/2019	533
Ammersee	A20	DE	47.99891	11.16727	01/07/2019	533
Ammersee	A3	DE	47.95090	11.15058	09/07/2019	533
Ammersee	A8	DE	47.96446	11.10731	09/07/2019	533
Aiguebelette	Ai1	FR	45.57211	5.79848	10/09/2018	390
Aiguebelette	Ai10	FR	45.54199	5.78049	10/09/2018	390
Aiguebelette	Ai11	FR	45.54100	5.78173	10/09/2018	390
Aiguebelette	Ai12	FR	45.54014	5.78669	10/09/2018	390
Aiguebelette	Ai13	FR	45.54211	5.79832	10/09/2018	390
Aiguebelette	Ai14	FR	45.54189	5.80484	10/09/2018	390
Aiguebelette	Ai15	FR	45.54246	5.81133	10/09/2018	390
Aiguebelette	Ai16	FR	45.54378	5.81212	10/09/2018	390
Aiguebelette	Ai17	FR	45.55242	5.80903	10/09/2018	390
Aiguebelette	Ai18	FR	45.55840	5.80499	10/09/2018	390
Aiguebelette	Ai19	FR	45.56690	5.80855	10/09/2018	390
Aiguebelette	Ai2	FR	45.57191	5.79675	10/09/2018	390
Aiguebelette	Ai20	FR	45.57502	5.80528	10/09/2018	390
Aiguebelette	Ai21	FR	45.57497	5.80231	10/09/2018	390
Aiguebelette	Ai3	FR	45.56791	5.79544	10/09/2018	390
Aiguebelette	Ai4	FR	45.56566	5.79622	10/09/2018	390
Aiguebelette	Ai5	FR	45.56089	5.79506	10/09/2018	390
Aiguebelette	Ai6	FR	45.55620	5.79524	10/09/2018	390
Aiguebelette	Ai7	FR	45.55269	5.79172	10/09/2018	390
Aiguebelette	Ai8	FR	45.54792	5.78324	10/09/2018	390
Aiguebelette	Ai9	FR	45.54327	5.77618	10/09/2018	390
Bourget	B1	FR	45.65583	5.86818	03/10/2018	232
Bourget	B10	FR	45.66459	5.85908	03/10/2018	232
Bourget	B10	FR	45.66459	5.85908	03/10/2018	232
Bourget	B11	FR	45.67581	5.85749	03/10/2018	232
Bourget	B12	FR	45.68594	5.85865	03/10/2018	232
Bourget	B12	FR	45.68594	5.85865	03/10/2018	232
Bourget	B1	FR	45.65583	5.86818	03/10/2018	232
Bourget	B2	FR	45.65182	5.87566	03/10/2018	232
Bourget	B2	FR	45.65182	5.87566	03/10/2018	232
Bourget	B3	FR	45.65204	5.88498	03/10/2018	232
Bourget	B3	FR	45.65204	5.88498	03/10/2018	232
Bourget	B4	FR	45.65437	5.89284	03/10/2018	232
Bourget	B4	FR	45.65437	5.89284	03/10/2018	232
Bourget	B5	FR	45.66486	5.89462	03/10/2018	232
Bourget	B5	FR	45.66486	5.89462	03/10/2018	232
Bourget	B6	FR	45.67652	5.89141	03/10/2018	232
Bourget	B6	FR	45.67652	5.89141	03/10/2018	232
Bourget	B7	FR	45.68807	5.89355	03/10/2018	232
Bourget	B7	FR	45.68807	5.89355	03/10/2018	232
Bourget	B8	FR	45.65624	5.86561	03/10/2018	232
Bourget	B8	FR	45.65624	5.86561	03/10/2018	232
Bourget	B9	FR	45.65929	5.85921	03/10/2018	232
Bourget	B9	FR	45.65929	5.85921	03/10/2018	232
Garda	CAS	IT	45.73623	10.79145	19/08/2019	65
Garda	CAS	IT	45.73623	10.79145	19/08/2019	65
Lugano	CHST1	CH	45.98657	8.95634	20/09/2019	271
Lugano	CHST10	CH	45.98921	8.98905	11/10/2019	271
Lugano	CHST10	CH	45.98921	8.98905	11/10/2019	271
Lugano	CHST1	CH	45.98657	8.95634	20/09/2019	271
Lugano	CHST2	CH	45.99841	8.94819	20/09/2019	271
Lugano	CHST2	CH	45.99841	8.94819	20/09/2019	271
Lugano	CHST3	CH	46.00319	8.96056	23/10/2019	271
Lugano	CHST3	CH	46.00319	8.96056	23/10/2019	271
Lugano	CHST4	CH	46.00234	8.96157	23/10/2019	271
Lugano	CHST4	CH	46.00234	8.96157	23/10/2019	271
Lugano	CHST5	CH	46.00408	8.99541	24/09/2019	271
Lugano	CHST5	CH	46.00408	8.99541	24/09/2019	271
Lugano	CHST6	CH	46.01544	9.02005	26/09/2019	271
Lugano	CHST6	CH	46.01544	9.02005	26/09/2019	271
Lugano	CHST7	CH	46.03052	9.12308	08/10/2019	271
Lugano	CHST7	CH	46.03052	9.12308	08/10/2019	271
Lugano	CHST8	CH	46.03010	9.12278	08/10/2019	271
Lugano	CHST8	CH	46.03010	9.12278	08/10/2019	271
Lugano	CHST9	CH	45.99170	8.99942	11/10/2019	271
Lugano	CHST9	CH	45.99170	8.99942	11/10/2019	271
Garda	CISA	IT	45.53159	10.72594	19/08/2019	65
Garda	CISA	IT	45.53159	10.72594	19/08/2019	65
Faaker	Faak1	AT	46.56965	13.92774	16/09/2019	554
Faaker	Faak2	AT	46.57522	13.93253	16/09/2019	554
Faaker	Faak3	AT	46.57573	13.92676	16/09/2019	554
Garda	GAR	IT	45.62537	10.57658	03/07/2019	65
Garda	GAR	IT	45.62537	10.57658	03/07/2019	65
Garda	GOL	IT	45.84395	10.82783	19/08/2019	65
Garda	GOL	IT	45.84395	10.82783	19/08/2019	65
Garda	MON	IT	45.52448	10.54645	03/07/2019	65
Mondsee	Mon1	AT	47.80826	13.41693	12/10/2018	481
Mondsee	Mon10	AT	47.84562	13.37172	11/10/2018	481
Mondsee	Mon10	AT	47.84562	13.37172	11/10/2018	481
Mondsee	Mon1	AT	47.80826	13.41693	12/10/2018	481
Mondsee	Mon2	AT	47.79940	13.42248	11/10/2018	481
Mondsee	Mon2	AT	47.79940	13.42248	11/10/2018	481
Mondsee	Mon3	AT	47.80140	13.43838	11/10/2018	481
Mondsee	Mon3	AT	47.80140	13.43838	11/10/2018	481
Mondsee	Mon4	AT	47.82743	13.39087	11/10/2018	481
Mondsee	Mon4	AT	47.82743	13.39087	11/10/2018	481
Mondsee	Mon5	AT	47.83392	13.36548	12/10/2018	481
Mondsee	Mon5	AT	47.83392	13.36548	12/10/2018	481
Mondsee	Mon6	AT	47.84055	13.34793	12/10/2018	481
Mondsee	Mon6	AT	47.84055	13.34793	12/10/2018	481
Mondsee	Mon7	AT	47.82017	13.39128	12/10/2018	481
Mondsee	Mon7	AT	47.82017	13.39128	12/10/2018	481
Mondsee	Mon8	AT	47.81693	13.36557	12/10/2018	481
Mondsee	Mon8	AT	47.81693	13.36557	12/10/2018	481
Mondsee	Mon9	AT	47.84970	13.34767	12/10/2018	481
Mondsee	Mon9	AT	47.84970	13.34767	12/10/2018	481
Garda	MON	IT	45.52448	10.54645	03/07/2019	65
Garda	PAC	IT	45.46369	10.71086	20/08/2019	65
Garda	PAC	IT	45.46369	10.71086	20/08/2019	65
Garda	RIV	IT	45.87781	10.85537	19/08/2019	65
Garda	RIV	IT	45.87781	10.85537	19/08/2019	65
Bled	Sample1	SI	46.36191	14.08485	12/08/2019	475
Bled	Sample2	SI	46.35919	14.08549	12/08/2019	475
Bled	Sample3	SI	46.36294	14.09024	12/08/2019	475
Bled	Sample4	SI	46.36269	14.08961	12/08/2019	475
Bled	Sample5	SI	46.36254	14.09943	12/08/2019	475
Bled	Sample6	SI	46.36373	14.10294	12/08/2019	475
Bled	Sample7	SI	46.36503	14.10622	12/08/2019	475
Bled	Sample8	SI	46.36878	14.10787	12/08/2019	475
Bled	Sample9	SI	46.36633	14.08609	12/08/2019	475
Staffelsee	SF12	DE	47.69782	11.17805	08/07/2019	649
Staffelsee	SF14	DE	47.70164	11.15929	08/07/2019	649
Staffelsee	SF2a	DE	47.67812	11.15076	08/07/2019	649
Staffelsee	SF5a	DE	47.69153	11.15798	08/07/2019	649
Staffelsee	SF8a	DE	47.70493	11.17416	08/07/2019	649
Staffelsee	SF9	DE	47.68980	11.17914	08/07/2019	649
Garda	SIR1	IT	45.47279	10.60052	03/07/2019	65
Garda	SIR1	IT	45.47279	10.60052	03/07/2019	65
Garda	SIR2	IT	45.46284	10.62006	03/07/2019	65
Garda	SIR2	IT	45.46284	10.62006	03/07/2019	65
Starnberger_See	T1	DE	47.87994	11.28782	18/07/2019	584
Starnberger_See	T10	DE	47.98122	11.33359	19/07/2019	584
Starnberger_See	T10	DE	47.98122	11.33359	19/07/2019	584
Starnberger_See	T11	DE	47.99569	11.34602	19/07/2019	584
Starnberger_See	T11	DE	47.99569	11.34602	19/07/2019	584
Starnberger_See	T13	DE	47.98629	11.35849	19/07/2019	584
Starnberger_See	T13	DE	47.98629	11.35849	19/07/2019	584
Starnberger_See	T14	DE	47.97844	11.35676	19/07/2019	584
Starnberger_See	T14	DE	47.97844	11.35676	19/07/2019	584
Starnberger_See	T15	DE	47.96721	11.34848	19/07/2019	584
Starnberger_See	T15	DE	47.96721	11.34848	19/07/2019	584
Starnberger_See	T16	DE	47.95891	11.34504	19/07/2019	584
Starnberger_See	T16	DE	47.95891	11.34504	19/07/2019	584
Starnberger_See	T17	DE	47.94940	11.34139	19/07/2019	584
Starnberger_See	T17	DE	47.94940	11.34139	19/07/2019	584
Starnberger_See	T18	DE	47.93047	11.33244	19/07/2019	584
Starnberger_See	T18	DE	47.93047	11.33244	19/07/2019	584
Starnberger_See	T19	DE	47.91982	11.33046	19/07/2019	584
Starnberger_See	T19	DE	47.91982	11.33046	19/07/2019	584
Starnberger_See	T1	DE	47.87994	11.28782	18/07/2019	584
Starnberger_See	T2	DE	47.88604	11.28017	18/07/2019	584
Starnberger_See	T20	DE	47.90935	11.33329	18/07/2019	584
Starnberger_See	T20	DE	47.90935	11.33329	18/07/2019	584
Starnberger_See	T21	DE	47.89468	11.33405	18/07/2019	584
Starnberger_See	T21	DE	47.89468	11.33405	18/07/2019	584
Starnberger_See	T22	DE	47.88537	11.33308	18/07/2019	584
Starnberger_See	T22	DE	47.88537	11.33308	18/07/2019	584
Starnberger_See	T23	DE	47.86901	11.33393	18/07/2019	584
Starnberger_See	T23	DE	47.86901	11.33393	18/07/2019	584
Starnberger_See	T24	DE	47.85453	11.33647	18/07/2019	584
Starnberger_See	T24	DE	47.85453	11.33647	18/07/2019	584
Starnberger_See	T25	DE	47.84118	11.33833	18/07/2019	584
Starnberger_See	T25	DE	47.84118	11.33833	18/07/2019	584
Starnberger_See	T27	DE	47.82074	11.31834	18/07/2019	584
Starnberger_See	T27	DE	47.82074	11.31834	18/07/2019	584
Starnberger_See	T28	DE	47.82760	11.29832	18/07/2019	584
Starnberger_See	T28	DE	47.82760	11.29832	18/07/2019	584
Starnberger_See	T2	DE	47.88604	11.28017	18/07/2019	584
Starnberger_See	T3	DE	47.89441	11.27227	18/07/2019	584
Starnberger_See	T30	DE	47.85375	11.29653	18/07/2019	584
Starnberger_See	T30	DE	47.85375	11.29653	18/07/2019	584
Starnberger_See	T3	DE	47.89441	11.27227	18/07/2019	584
Starnberger_See	T4	DE	47.90609	11.27956	18/07/2019	584
Starnberger_See	T4	DE	47.90609	11.27956	18/07/2019	584
Starnberger_See	T5	DE	47.93167	11.29896	19/07/2019	584
Starnberger_See	T5	DE	47.93167	11.29896	19/07/2019	584
Starnberger_See	T6	DE	47.94164	11.31050	19/07/2019	584
Starnberger_See	T6	DE	47.94164	11.31050	19/07/2019	584
Starnberger_See	T7	DE	47.95147	11.31009	19/07/2019	584
Starnberger_See	T7	DE	47.95147	11.31009	19/07/2019	584
Starnberger_See	T8	DE	47.96140	11.31807	19/07/2019	584
Starnberger_See	T8	DE	47.96140	11.31807	19/07/2019	584
Starnberger_See	T9	DE	47.96926	11.32602	19/07/2019	584
Starnberger_See	T9	DE	47.96926	11.32602	19/07/2019	584
Garda	TRE	IT	45.75651	10.75008	03/07/2019	65
Garda	TRE	IT	45.75651	10.75008	03/07/2019	65
Lac des Confins	LA1.1	FR	45.91690	6.47664	22/07/2013	1355
Lac des Confins	LA1.2	FR	45.91512	6.47567	22/07/2013	1355
Lac Achard	LA10.1	FR	45.11327	5.90188	10/07/2013	1917
Lac Achard	LA10.2	FR	45.11369	5.90116	10/07/2013	1917
Lac Achard	LA10.3	FR	45.11511	5.90196	10/07/2013	1917
Lac Robert Sud‐Est	LA11.1	FR	45.13146	5.91827	11/07/2013	1998
Lac Robert Sud‐Est	LA11.2	FR	45.13078	5.91752	11/07/2013	1998
Lac Robert Sud‐Est	LA11.3	FR	45.13077	5.91880	11/07/2013	1998
petit Lac Bernard, au sud ouest du Lac Longuet	LA13.1	FR	45.15272	5.93420	17/07/2013	2000
petit Lac Bernard, au sud ouest du Lac Longuet	LA13.2	FR	45.15222	5.93397	17/07/2013	2000
petit Lac Bernard, au sud ouest du Lac Longuet	LA13.3	FR	45.15249	5.93332	17/07/2013	2000
Lac Longet de Belledonne	LA14.1	FR	45.15244	5.93547	13/07/2013	2027
Lac Longet de Belledonne	LA14.2	FR	45.15368	5.93778	13/07/2013	2027
Lac Longet de Belledonne	LA14.3	FR	45.15280	5.93759	13/07/2013	2027
Lac David	LA15.1	FR	45.15087	5.94320	17/07/2013	2212
Lac David	LA15.2	FR	45.15047	5.94195	17/07/2013	2212
Lac David	LA15.3	FR	45.15010	5.94286	17/07/2013	2212
Lac Merlat	LA16.1	FR	45.15848	5.93099	14/07/2013	2044
Lac Merlat	LA16.2	FR	45.15833	5.93335	14/07/2013	2044
Lac Merlat	LA16.3	FR	45.15758	5.93121	14/07/2013	2044
Lac Claret	LA17.1	FR	45.15637	5.94292	17/07/2013	2045
Lac Claret	LA17.2	FR	45.15553	5.94203	14/07/2013	2045
Lac Claret	LA17.3	FR	45.15523	5.94292	14/07/2013	2045
Lac Blanc de l'Herpie	LA18.1	FR	45.11179	6.10397	24/07/2013	2525
Lac Blanc de l'Herpie	LA18.2	FR	45.11718	6.10628	24/07/2013	2525
Lac Blanc de l'Herpie	LA18.3	FR	45.11497	6.10832	24/07/2013	2525
Lac Noir de Poutran	LA19.1	FR	45.11692	6.08484	23/07/2013	2047
Lac Noir de Poutran	LA19.2	FR	45.11747	6.08543	23/07/2013	2047
Lac Besson‐Rond	LA20.1	FR	45.67938	6.85323	23/07/2013	2070
Lac Besson‐Rond	LA20.3	FR	45.67852	6.85216	23/07/2013	2070
Lac de Presset	LA24.1	FR	45.65022	6.66482	20/07/2013	2514
Lac de Presset	LA24.2	FR	45.64934	6.66396	20/07/2013	2514
Lac de Presset	LA24.3	FR	45.64905	6.66465	20/07/2013	2514
Lac Sans Fond	LA29.1	FR	45.67608	6.88848	14/07/2013	2456
Lac Sans Fond	LA29.2	FR	45.67457	6.88663	14/07/2013	2456
Lac Sans Fond	LA29.3	FR	45.67417	6.88785	14/07/2013	2456
Lac Longet du petit saint bernard	LA30.1	FR	45.67602	6.88821	15/07/2013	2316
Lac Longet du petit saint bernard	LA30.2	FR	45.67397	6.88663	15/07/2013	2316
Lac Longet du petit saint bernard	LA30.3	FR	45.67449	6.88856	15/07/2013	2316
Lac de la Plagne	LA31.1	FR	45.48579	6.84170	11/08/2013	2145
Lac de la Plagne	LA31.2	FR	45.48423	6.84021	11/08/2013	2145
Lac de la Plagne	LA31.3	FR	45.48318	6.84293	11/08/2013	2145
Grand lac du Chardonnet	LA32.1	FR	45.46612	6.88511	16/08/2013	2384
Grand lac du Chardonnet	LA32.3	FR	45.46555	6.88238	16/08/2013	2384
Lac du Grataleu	LA33.1	FR	45.46186	6.85849	16/08/2013	2512
Lac du Grataleu	LA33.3	FR	45.46097	6.86004	16/08/2013	2512
Lac Brulet d'archebroc	LA35.2	FR	45.57395	6.96936	22/08/2013	2697
Lac Brulet d'archebroc	LA35.3	FR	45.57322	6.96995	22/08/2013	2697
Lac Noir du clou	LA37.1	FR	45.56956	6.98395	22/08/2013	2618
Lac Noir du clou	LA37.2	FR	45.57111	6.98449	22/08/2013	2618
Lac Noir du clou	LA37.3	FR	45.56950	6.98671	22/08/2013	2618
Lac Noir du Carro	LA38.1	FR	45.42187	7.12494	12/08/2013	2750
Lac Noir du Carro	LA38.2	FR	45.42073	7.12434	12/08/2013	2750
Lac Blanc du Carro	LA39.1	FR	45.42139	7.12742	13/08/2013	2753
Lac Blanc du Carro	LA39.2	FR	45.42020	7.12597	13/08/2013	2753
Lac Blanc du Carro	LA39.3	FR	45.41983	7.12750	13/08/2013	2753
Lac des Evettes	LA40.1	FR	45.35593	7.11486	05/08/2013	2539
Lac des Evettes	LA40.2	FR	45.35506	7.11653	05/08/2013	2539
Lac des Evettes	LA40.3	FR	45.35455	7.11477	05/08/2013	2539
Lac de la Leisse (sud)	LA41.1	FR	45.42002	6.91068	14/08/2013	2798
Lac de la Leisse (sud)	LA41.2	FR	45.41871	6.91038	14/08/2013	2798
Lac de la Leisse (sud)	LA41.3	FR	45.41843	6.91115	14/08/2013	2798
Lac des Nettes	LA42.2	FR	45.41367	6.90014	14/08/2013	2641
Lac des Nettes	LA42.3	FR	45.41288	6.89832	14/08/2013	2641
Lac Long	LA43.1	FR	45.39696	6.79092	16/08/2013	2467
Lac Long	LA43.2	FR	45.39618	6.78960	16/08/2013	2467
Lac Long	LA43.3	FR	45.39574	6.79153	16/08/2013	2467
Lac Rond	LA44.1	FR	45.38880	6.79961	16/08/2013	2500
Lac Rond	LA44.3	FR	45.38804	6.80104	16/08/2013	2500
Lac du Pelve	LA45.1	FR	45.36081	6.80098	20/08/2013	2574
Lac du Pelve	LA45.2	FR	45.36181	6.80091	20/08/2013	2574
Lac de Roche ferran	LA46.1	FR	45.35883	6.79230	21/08/2013	2619
Lac de Roche ferran	LA46.2	FR	45.36030	6.79214	21/08/2013	2619
Lac de Roche ferran	LA46.3	FR	45.36105	6.79541	20/08/2013	2619
Plan du lac	LA48.1	FR	45.33839	6.83222	07/08/2013	2362
Plan du lac	LA48.3	FR	45.33622	6.83174	07/08/2013	2362
Lac de l'Arpont	LA49.1	FR	45.31523	6.77605	28/08/2013	2666
Lac de l'Arpont	LA49.2	FR	45.31665	6.77441	28/08/2013	2666
Lac de l'Arpont	LA49.3	FR	45.31621	6.77667	28/08/2013	2666
Lac de Lessy	LA5.1	FR	45.98818	6.44088	23/07/2013	1735
Lac de Lessy	LA5.2	FR	45.98814	6.43792	23/07/2013	1735
Lac de Lessy	LA5.3	FR	45.98658	6.43613	23/07/2013	1735
Lac inférieur de Lanserlia	LA50.1	FR	45.33361	6.85767	22/08/2013	2745
Lac inférieur de Lanserlia	LA50.2	FR	45.33441	6.85746	22/08/2013	2745
Lac inférieur de Lanserlia	LA50.3	FR	45.33429	6.85885	28/08/2013	2745
Lac de la Leisse (milieu)	LA51.1	FR	45.42273	6.91271	14/08/2013	2769
Lac de la Leisse (milieu)	LA51.2	FR	45.42211	6.91262	14/08/2013	2769
Lac de la Leisse (milieu)	LA51.3	FR	45.42184	6.91404	14/08/2013	2769
Lac supérieur de Lanserlia	LA52.1	FR	45.33680	6.85514	22/08/2013	2760
Lac supérieur de Lanserlia	LA52.2	FR	45.33748	6.85477	22/08/2013	2760
Lac supérieur de Lanserlia	LA52.3	FR	45.33740	6.85605	22/08/2013	2760
Lac des Cerces	LA53.1	FR	45.07238	6.47373	23/07/2013	2410
Lac des Cerces	LA53.2	FR	45.07073	6.47352	23/07/2013	2410
Lac des Cerces	LA53.3	FR	45.07073	6.47596	23/07/2013	2410
Lac du Grand Ban	LA55.2	FR	45.08600	6.47591	23/07/2013	2465
Lac du Grand Ban	LA55.3	FR	45.08550	6.47217	23/07/2013	2465
Lac du Serpent	LA56.1	FR	45.05269	6.56178	31/07/2013	2448
Lac du Serpent	LA56.2	FR	45.05205	6.56346	31/07/2013	2448
Lac du Serpent	LA56.3	FR	45.05087	6.56198	31/07/2013	2448
Lac Laramon	LA57.1	FR	45.04934	6.55599	31/07/2013	2359
Lac Laramon	LA57.2	FR	45.04855	6.55444	31/07/2013	2359
Lac Laramon	LA57.3	FR	45.04786	6.55643	31/07/2013	2359
Lac Rond des Rochilles	LA58.1	FR	45.08667	6.48123	23/07/2013	2450
Lac Rond des Rochilles	LA58.2	FR	45.08525	6.48425	23/07/2013	2450
Lac Rond des Rochilles	LA58.3	FR	45.08470	6.48112	23/07/2013	2450
Lac des Béraudes	LA59.1	FR	45.58606	6.50357	19/07/2013	2504
Lac des Béraudes	LA59.2	FR	45.58592	6.50405	19/07/2013	2504
Lac des Béraudes	LA59.3	FR	45.58580	6.50368	19/07/2013	2504
Lac Sainte Marguerite du Thabor le plus haut	LA60.1	FR	45.12520	6.59439	25/07/2013	2513
Lac Sainte Marguerite du Thabor le plus haut	LA60.2	FR	45.12553	6.59282	25/07/2013	2513
Lac Sainte Marguerite du Thabor le plus haut	LA60.3	FR	45.12662	6.59363	25/07/2013	2513
Lac des Muandes (Névaches)	LA62.1	FR	45.08839	6.54384	01/08/2013	2387
Lac des Muandes (Névaches)	LA62.2	FR	45.08824	6.54483	01/08/2013	2387
Lac des Muandes (Névaches)	LA62.3	FR	45.08768	6.54400	01/08/2013	2387
Lac Blanc de Polset	LA63.1	FR	45.29405	6.64986	26/07/2013	2433
Lac Blanc de Polset	LA63.2	FR	45.29434	6.65388	26/07/2013	2433
Lac Blanc de Polset	LA63.3	FR	45.29342	6.65308	26/07/2013	2433
Lac inférieur du Merlet	LA64.1	FR	45.35848	6.64797	20/07/2013	2391
Lac inférieur du Merlet	LA64.2	FR	45.35779	6.64904	20/07/2013	2391
Lac inférieur du Merlet	LA64.3	FR	45.35753	6.64701	20/07/2013	2391
Lac de la Partie	LA66.1	FR	45.25788	6.66348	17/07/2013	2458
Lac de la Partie	LA66.2	FR	45.25896	6.66321	17/07/2013	2458
Lac du Mont Charvin	LA9.1	FR	45.80901	6.42210	16/07/2013	2011
Lac du Mont Charvin	LA9.2	FR	45.80833	6.42304	16/07/2013	2011
Lac du Mont Charvin	LA9.3	FR	45.80895	6.42345	16/07/2013	2011

*Note:* Complete metadata (with additional physical and chemical data) are given in Supporting Information at https://doi.org/10.57745/0TVDYI.

### 2.2 DNA Extraction and Sequencing

We extracted DNA contained in biofilm samples from each lake following the protocol proposed by Rivera et al. (2020). Briefly, the suspended diatom biofilm (2 mL) was centrifuged for 30 min at 13,000 rpm to recover the supernatant from which DNA was extracted using the NucleoSpin Soil kit (Macheray‐Nagel) following the manufacturer's protocol. DNA quantity and quality were checked using a NanoDrop 1000 Spectrophotometer (Thermo Fisher Scientific). For PCR, we amplified a 263 bp barcode of chloroplastic DNA, embedded in the rbcL gene, which encodes for ribulose‐1,5‐bisphosphate carboxylase/oxygenase. This barcode is recommended for diatom species identification (e.g., Kermarrec et al. 2013; Maitland et al. 2020). We used the diatom‐specific primers Diat_rbcL_708F_1 (AGGTGAAGTAAAAGGTTCWTACTTAAA), Diat_rbcL_708F_2 (AGGTGAAGTTAAAGGTTCWTAYTTAAA), and Diat_rbcL_708F_3 (AGGTGAAACTAAAGGTTCWTACTTAAA) as forward primers and Diat_rbcL_R3_1 (CCTTCTAATTTACCWACWACTG) and Diat_rbcL_R3_2 (CCTTCTAATTTACCWACAACAG) as reverse primers (Vasselon et al. 2017). Each DNA extract was amplified in triplicate using equimolar mixes of the three forward and two reverse primers. Additionally, blank samples using water of molecular quality were run in parallel to check for potential contaminations introduced during handling the samples in the lab. The DNA extracts were used as templates for polymerase chain reaction amplification (PCR) from a final volume of 25 μL following mix and thermal cycling conditions used by Rivera et al. (2020). Thermal cycling conditions consisted of 33 cycles of 1 min at 95°C, 1 min at 54°C, and 1 min at 72°C. For each sample, PCR products from the three replicates were pooled, and 50 μL was sent for sequencing to a genomic platform, using MiSeq technology (Illumina) and the V1 reagent kit (2 × 250 bp). Four different runs were carried out at two different platforms: 3 at the GetPlage platform (Toulouse, France, https://get.genotoul.fr), and one at the University of Geneva for Lake Geneva samples (https://id‐gene.com).

### 2.3 Bioinformatic Analysis

To describe diatom diversity, the demultiplexed reads were analyzed with a pipeline combining Cutadapt v3.5 (Martin 2011) to remove primer sequences and DADA2 (Callahan et al. 2016) to delineate ASVs. According to the quality profiles, forward and reverse reads were trimmed at 200 and 170 nucleotides, respectively. Truncated sequences were filtered out using the *filterAndTrim()* function from the *dada2* R‐package (Callahan et al. 2016) when they contained ambiguous nucleotides or exceeded a maximum expected error number (MaxEE) greater than two. After applying the DADA2 denoising model to determine an error model for each run using the *learnErrors()* function, the forward and reverse reads were merged to obtain the full denoised sequences using the *dada()* function. The preceding steps were applied separately for each sequencing run, which were then merged using the *mergeSequenceTables()* function, and chimeras were removed using the *removeBimeraDenovo()* function.

In the total ASV table, ASVs not matching the target length (263 bp) or with fewer than 10 reads were removed. Since rbcL is a coding region, we used MEGAX (Kumar et al. 2018) to check for indels and stop codons, without finding any. We assigned taxonomy to ASVs using Diat.barcode reference library version 11 (Rimet, Gusev, et al. 2019) with a minimum bootstrap confidence of 60%, and non‐Bacillariophyta ASVs were excluded. Samples with fewer than 10,000 reads were removed (8 out of 514), and data were rarefied to 10,958 reads (the lowest number of reads) using the *vegan* R‐package (Oksanen et al. 2025). The final database includes 2523 ASVs (1444 in high altitude, 1507 in lowland) and 514 samples, totaling 5,632,412 reads.

### 2.4 Statistical Analyses

#### 2.4.1 Changes in Shared Taxa and Beta‐Diversity With Geographical Distance in Lowland and High‐Altitude Lakes

Before analyzing shared taxa between samples, we compared alpha diversities between high‐altitude and lowland lake samples with the Kruskal‐Wallis test for richness with the vegan R‐package (Oksanen et al. 2025) and the Hutcheson *t*‐test for the Shannon index with the ecolTest R‐package (Salinas 2021).

We calculated the Jaccard's index (Jaccard 1901) to measure the proportion of ASVs shared between samples from different lakes for high‐altitude and lowland lakes (excluding comparisons between the two groups) using the *dist.ldc()* function of the adespatial R‐package (Dray et al. 2025). Index values were grouped into seven classes of geographical distance between samples: < 200 m, [200 m, 2 km], [2 km, 20 km], [20 km, 50 km], [50 km, 100 km], [100 km, 200 km], [200 km, 654 km].

For each geographical distance class, a Wilcoxon rank sum test was carried out to test the difference between Jaccard index values between high‐altitude and lowland lakes. Kruskal‐Wallis and Dunn tests were calculated to compare indices between class distances using PAST software (Hammer et al. 2001). An NMDS (Non‐metric multimetric dimensional scaling) was calculated using the *metaMDS()* function from the *vegan* R‐package based on Jaccard distance to visualize the beta‐diversities of lowland and high‐altitude lake communities.

#### 2.4.2 Correlations Between Lake Diversity and Lake Surface and Watershed Size

We checked for a relationship between lake, watershed surface, and lake richness (total number of ASVs in each lake) using the “Non‐linear fit” option of Past software.

In addition, species accumulation curves were drawn for both high‐altitude and lowland lakes by merging samples from the same lake into a single community and summing ASV reads across samples. Accumulation curves were estimated using the *specaccum()* function (the random permutation method and the other default arguments were used), and the Chao2 (Chao 1987) index (asymptotic species richness) was calculated with the *specpool()* function for high‐altitude and lowland lakes with the *vegan* R‐package.

#### 2.4.3 Evaluation of Assembly Processes and Environmental Factors Shaping Diatom Communities in High‐Altitude and Lowland Lakes

Assembly processes were assessed relative to phylogenetic and taxonomic turnover according to Stegen et al. (2013) analytical framework. Briefly, this framework compares in a 1st step phylogenetic turnover of samples' communities to the null model in order to assess selection processes. If communities are significantly phylogenetically over‐clustered, environmental filtering is interpreted as the dominant process for these samples. Conversely, if they are significantly over‐dispersed, competition is interpreted as the dominant structuring process. Samples that do not differ significantly from the null model are tested in a 2nd step. This 2nd step compares taxonomic turnover to the null model to assess dispersal processes. If sample turnover is significantly higher than expected by chance, this is interpreted as a dispersal limitation, whereas if it is smaller than expected by chance, this is interpreted as dispersal homogenization. For the other samples, which do not differ from the null model, stochastic processes are considered to be dominant.

To this end, a reference phylogeny was constructed using long sequences from the reference library Diat.barcode v12 (Rimet, Gusev, et al. 2019), and an alignment was performed on 1046 nucleotides and 3506 sequences. The construction of this reference phylogeny was based on a maximum likelihood algorithm using raxml‐8.2.12 software (Stamatakis 2014) with 100 rapid bootstrap replicates and the GTRGamma model. The phylogeny is provided as Supporting Information S2. ASVs were aligned in a FASTA file and placed in this reference phylogeny using RaxmlGUI (Silvestro and Michalak 2012) with the “enforce constrain mode” and the “backbone option” (Supporting Information S3). This enables getting the phylogenetic distances between ASVs.

Assembly processes were estimated using the *microeco* R‐package (Liu et al. 2021). To analyze processes between lakes, samples from the same lake were merged: reads were summed per ASV, averages were calculated for water chemical measurements (pH, conductivity, nitrate, total phosphorus) and geographical coordinates, and lake morphometric measures (lake surface, depth, altitude, residence time, basin surface) remained unchanged. At this stage, the phylogenetic signal was tested for chemical parameters with a Mantel correlogram using the *cal_mantel_corr()* function and default options. This step is essential since a phylogenetic signal conditions the use of NTI indices (Nearest Taxon Index) to estimate assemblage processes. The global dataset was divided into two according to lake type: high‐altitude and lowland lakes. Following the approach of Stegen et al. (2013), selection processes are estimated from null models based on betaNTI (beta‐nearest taxon index) distances, calculated between lakes using the ASV table and the phylogenetic distances between ASVs. To assess dispersal processes, null models based on the Raup‐Crick metric using Bray‐Curtis dissimilarities (RCbray) are calculated between lakes based on the ASV table using the *cal_rcbray()* function and 1000 runs. These processes were estimated separately for high‐altitude and lowland lakes. Then we evaluated the proportion of the inferred processes using the *cal_process()* function.

After this first stage of analyses, we quantified the variance of diatom communities explained by the three classes of environmental factors (chemical, hydro‐morphological, and spatial) using a variance partitioning analysis and the *varpart()* function of the *vegan* R‐package. We performed this analysis for both lake types separately. Chemical and hydro‐morphometric data were standardized (mean and variance respectively equal 0 and 1). Spatial parameters were estimated using the approach of principal coordinate analysis of neighbor matrices (PCNM; Borcard and Legendre 2002) using the *pcnm()* function from the vegan R‐package), with the three first axes selected and then standardized. We performed a forward selection on the chemical, hydro‐morphometric, and spatial parameters separately to estimate the significant explicative variables of each using the *ordistep()* function of the vegan R‐package.

#### 2.4.4 Taxa Co‐Occurrence in High‐Altitude and Lowland Lakes

Between lakes, the number of samplings varied considerably, from two or three samples for high‐altitude lakes to 152 samples for Lake Geneva. The large number of samples taken in Lac Geneva may result in a high probability of having the same ASV co‐occurring together, compared with high‐altitude lakes. In order not to artificially increase the correlations between ASVs when lakes were sampled many times, samples from the same lake were merged into a single community, as done in § 2.4.3.

A co‐occurrence network was calculated for each type of lake, using the SparCC (Sparse Correlations for Compositional data) method and the *sparcc()* function from the SpiecEasi R‐package (Kurtz et al. 2015, 2025). We chose this method because it has been tailored to genomic data to infer co‐occurrence networks between taxa (or genes) from compositional data (Kurtz et al. 2015) and because it has been widely used with success for such kinds of data (Kajihara and Hynson 2024). In both co‐occurrence networks, only associations between ASVs with a correlation coefficient greater than 0.5 were retained as significant associations. The final co‐occurrence networks were visualized using the layout_on_grid() function from the igraph R‐package (Csardi and Nepusz 2006). We characterized the structure of networks by calculating their level of connectivity (i.e., number of connected ASVs divided by the total number of ASV in the network) and connectance (i.e., number of edges divided by the number of possible edges in the network), using the degree() function and the edge_density() function of the igraph R‐package, respectively.

## 3 Results

### 3.1 Changes in Shared Taxa and Beta‐Diversity With Geographical Distance in Lowland and High‐Altitude Lakes

**FIGURE 2:**
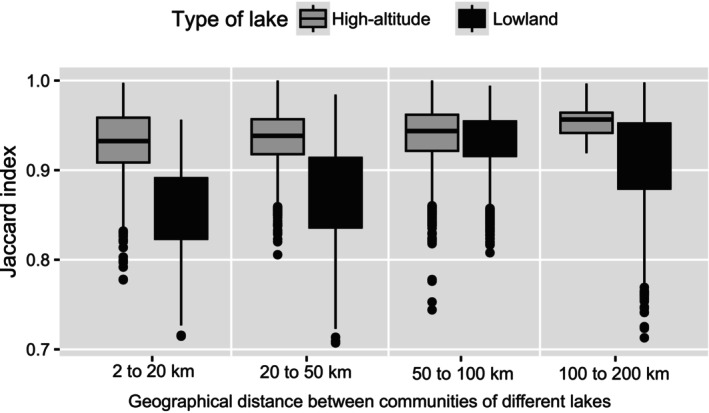
Similarity in terms of shared ASVs between communities of different lakes according to geographical distance. The Jaccard index measures the proportion of shared taxa between two samples. The indexes were calculated between communities of high‐altitude lakes and between communities of lowland lakes. The indices are presented in box‐plots according to classes of geographical distances between communities.

**FIGURE 3:**
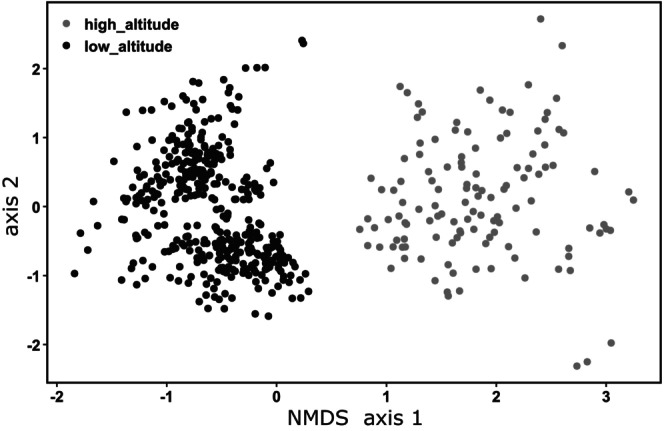
Non‐metric multidimensional scaling (NMDS) showing the similarity in terms of ASV composition between communities of high‐altitude and lowland lakes. The NMDS is based on the Jaccard distance matrix, stress: 0.191, *R* ^2^ = 0963 (non‐linear fit).

Samples from lowland lakes had significantly higher diversities than those from high‐altitude lakes (Shannon with Hutcheson t‐test and richness with Kruskal‐Wallis test: *p* < 2 × 10^−16^). Jaccard indices were consistently higher between groups of high‐altitude lakes compared to groups of lowland lakes, regardless of geographic distance class (Figure 2). Therefore, when considering two lakes separated by the same distance range, communities from lowland lakes share more ASVs than communities from high‐altitude lakes (*p* < 2 × 10^−16^ Wilcoxon test). Within each lake type, Jaccard indices increased with distance class, indicating that the most geographically distant lakes share fewer ASVs (Kruskall‐Wallis *p* < 0.005, and all pairwise Dunn tests *p* < 0.005). NMDS analysis based on ASV tables revealed clear differences between samples according to lake type (Figure 3). This difference was supported by the low overlap of ASVs, with only 17% shared between high‐altitude and lowland lakes, and 41% being specific to high‐altitude lakes and 42% specific to lowland lakes.

### 3.2 Correlations Between Lake Diversity With Lake Surface and Watershed Size

**FIGURE 4:**
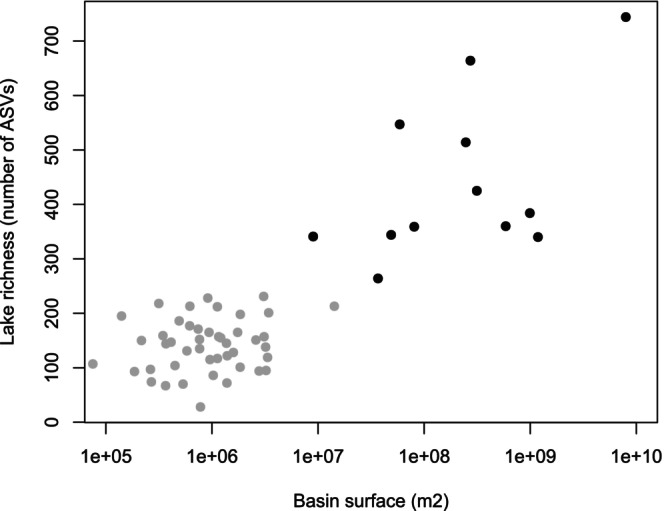
Relation between lake basin surface and lake richness. Lake richness is the total number of ASVs in a lake. Black dots represent lowland lakes, and gray dots high‐altitude lakes. Overall, relationship is significant between lake surface and ASV richness (logarithmic regression, r^2^ = 0.73, *p* < 0.005).

We found significant positive relationships (Spearman correlation test) between lake diversity (total number of ASVs in each lake) and both lake surface (*r* ^2^ = 0.73, *p* < 0.005) and watershed surface (*r* ^2^ = 0.70, *p* < 0.005), as shown in Figure 4 for watershed surface. However, these correlations were no longer significant when the tests were restricted to high‐altitude or lowland lakes.

In addition, lowland lakes showed a faster increasing accumulation curve than high‐altitude lakes and a higher cumulative number of ASVs (Supporting Information S4). Gamma diversities estimated by the Chao2 index are significantly higher in lowland lakes (2273 ASVs ±84 sd) than in high‐altitude lakes (2079 ASVs ±71 sd). This trend is supported by the observed richness, which is 1444 ASVs for high‐altitude lakes and 1507 ASVs for lowland lakes.

### 3.3 Evaluation of Assembly Processes and Environmental Factors Shaping Diatom Communities in High‐Altitude and Lowland Lakes

**FIGURE 5:**
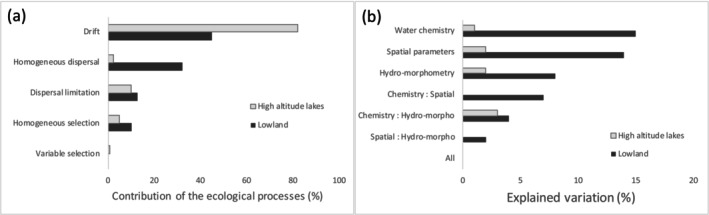
(a) Contribution of the ecological processes structuring diatom communities in high‐altitude and lowland lakes using Stegen et al. (2013) methodology. (b) Contribution of environmental (water chemistry, hydromorphometry) and dispersal filters (spatial distance) to explain communities variability using variance partitioning (unexplained variance: 92% for high‐altitude lakes and 50% for lowland lakes).

A significant positive correlation over short phylogenetic distances (Supporting Information S5) was observed for the tested environmental parameters (conductivity, pH, nitrates), indicating that phylogenetically neighboring taxa share similar environmental optima. This correlation supports the use of the betaNTI (measuring phylogenetic turnover) and RCbray (measuring taxonomic turnover) metrics as indicators of selection and dispersal processes, respectively. Null models based on these two metrics showed that ecological drift (i.e., taxonomic turnover of samples was not significantly different from the null model) was the overriding process for both lake types (high‐altitude lakes: 82%, lowland lakes: 45%), but to a lesser extent for lowland lakes (Figure 5a). Homogeneous selection (i.e., communities show significant higher phylogenetic over‐clustering than the null model) was similar between lowland and high‐altitude lakes (10% and 5%, respectively). Dispersal processes were more important for lowland lakes (45%) than for high‐altitude lakes (12%). They were driven by the homogenization dispersal; i.e., taxonomic turnover was smaller than the null model (lowland lakes: 32%, high‐altitude lakes: 2%). Dispersal limitation (i.e., taxonomic turnover was higher than the null model) was similar between both types of lake (13% and 10%, respectively).

For the variance partitioning analysis, forward selection retained as explanatory variables: total phosphorus, pH, and nitrate for chemistry; elevation, depth, and water residence time for hydro‐morphometry; and the first three PCNM axes for spatial parameters (Figure 5b). The analysis showed that environmental parameters explained 50% and 8% of community variability in lowland and high‐altitude lakes, respectively. When excluding spatial parameters (even simultaneous effects), the variability explained is reduced to 27% for lowland lakes and 6% for high‐altitude lakes.

### 3.4 Taxa Co‐Occurrence in High‐Altitude and Lowland Lakes

**FIGURE 6:**
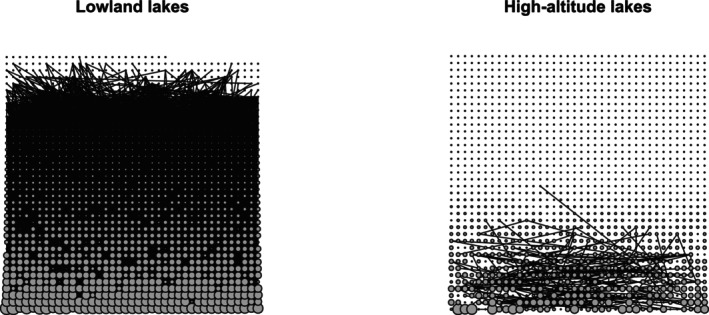
Co‐occurrence networks for lowland and high‐altitude lakes. A dot corresponds to an ASV, and an edge indicates a significant correlation between two ASVs above 0.5. ASVs are ordered by increasing abundance from the right bottom corner to the top left corner. Dot size corresponds to ASV reads sum in all samples.

Based on co‐occurrence networks, the number of co‐occurring ASVs was greater in lowland lakes than in high‐altitude lakes (Figure 6), indicating a more complex network in lowlands. This is supported by a stronger connectance in lowland lakes' network (edge density = 3.2 × 10^−2^) than in high‐altitude (edge density = 2 × 10^−4^), and the same trend is observed for connectivity (node density: high‐altitude lakes = 0.89, lowland lakes = 0.14).

## 4 Discussion

### 4.1 Stochastic Processes Are More Important in High‐Altitude Than in Lowland Lakes

Most of the samples (82%) from high‐altitude lakes showed community structures that did not deviate from those expected under the null model, compared with only 45% of samples from lowland lakes. Community structure in high‐altitude lakes was mainly driven by stochastic processes, as already observed (Ossyssek et al. 2023), unlike in lowland lakes. We can suppose that this difference between the two types of lakes is probably due to the extreme meteorological conditions in high‐altitude lakes, which create serious bottlenecks for diatom communities. Most of these lakes freeze for more than six months (Rivier 1997), which affects the survival of diatom cells trapped in the ice. Moreover, during the melting season, the massive influx of water from the melted snow and rain of the watershed causes strong turbulence and turbidity (Rimet, Feret, et al. 2019), which can strip biofilms where diatoms live and carry them out of the lake. These two phenomena are much less common in lowland lakes. However, when diatoms face unfavorable conditions, they are capable of producing resting spores (auxospores)—as most protists—and therefore should not be sensitive to bottlenecks. But less than 1% of vegetative cells are able to do so in freshwaters (Jewson 1992; Sutherland et al. 2023), and survival rates of spores are less than 40% in marine sediments (Sanyal et al. 2022). The production of resting spores is therefore a major bottleneck for diatoms, given the low probability of survival. These massive extinction/colonization processes largely favor stochastic processes (Vellend 2010) and are much more predominant in high‐altitude lakes than in lowland lakes.

### 4.2 Dispersal Processes Play Contrasting Roles in Lowland and in High‐Altitude Lakes

Dispersal processes play a major role in lowland and high‐altitude lakes. There are similarities in the way communities are structured by dispersal in high‐altitude and lowland lakes. For example, in both types of lake, we observed a decrease in the number of shared ASVs between lakes as the geographical distance between them increased. These results indicate that dispersal is limited, with around 10%–12% of the communities being shaped by this process. Such a decrease in community similarity with geographical distance has already been shown for river diatoms with morphological data in different regions of the world (Keck et al. 2018; Jamoneau et al. 2018; Wetzel et al. 2012) as well as on a global scale (Martín‐Devasa et al. 2024). However, large‐scale morphological databases suffer from a lack of taxonomic homogeneity due to varying identification skills between experts (Kahlert et al. 2009), a concern for biogeographical studies (Pinseel et al. 2024). The metabarcoding approach avoids this issue. This relationship between geographical distance and community similarity is now well admitted based on metabarcoding studies targeting microbial communities like protists (Lentendu and Dunthorn 2021) and bacteria (Trouche et al. 2021) in a variety of environments, including lakes' diatoms (Rimet, Pinseel, et al. 2023).

Beyond these similarities in the dispersal processes affecting communities living in high‐altitude and lowland lakes, certain specificities emerge depending on the type of lake. For example, for the same range of geographical distances, communities in high‐altitude lakes shared fewer ASVs than those in lowland lakes. Indeed, 32% of communities in lowland lakes show homogeneous dispersal (i.e., communities were more similar than expected by chance), compared with 2% in high‐altitude lakes. Homogenization in lowland lakes is a much stronger process than in high‐altitude lakes, which validates our hypothesis. We can suppose that the presence of numerous biotic (e.g., birds) and anthropogenic (human movement, boat transport, water ballast) vectors drives this homogenization process through dispersal. Dispersal of some freshwater diatom species by birds has already been proven experimentally (Faye et al. 2021), but it has also been observed in situ with *Silurus glanis* , an invasive fish in northern Italian rivers (Falasco et al. 2024), as well as via water ballast for some marine diatom species (MacGillivary and Kaczmarska 2015; Pfannkuchen et al. 2018). Such dispersal vectors are more seldom in high‐altitude.

A second explanation may be related to mass‐effect, gamma diversity, and lake size. Mass‐effect explains an important part of diatom communities in rivers (Leboucher et al. 2021) and lakes (Rimet, Canino, et al. 2023), and larger ecosystems provide a bigger target for dispersers than smaller ecosystems, which in turn increases their diversity and richness (Gilpin and Diamond 1976). We showed that there is a correlation between lake gamma diversity and lake surface as well as watershed surface (Figure 4). Moreover, in our study, surface areas and watersheds of lowland lakes were larger than those of high‐altitude lakes. Therefore, we can assume that mass‐effect may be of bigger importance in lowland lakes, which validates our 3rd hypothesis. Mass‐effect usually increases homogeneity between communities (Mouquet and Loreau 2003) and this homogenization has also been shown for river diatom communities (Bried and Vilmi 2022). Therefore, we can suppose that mass‐effect could explain why lowland lakes are more homogeneous with each other than high‐altitude lakes. However, this 2nd explanation needs to be tempered, as small lakes and watersheds were only found at high‐altitude, and large lakes and watersheds were found in the lowlands. Indeed, it is likely that the observed correlation between gamma diversity and lake or watershed size has a potential confounding influence with altitude.

The higher homogeneous dispersal processes observed in lowland lakes' communities can be part of the processes explaining the greater connectance and connectivity of lowland ecological networks compared to high‐altitude lakes (Figure 6). Analysis of ecological network topologies has been shown to be of great interest *because links* can be established with ecosystem resilience and resistance (e.g., Gao et al. 2022; Xu et al. 2022). In particular, ecosystems hosting ecological networks with higher connectance and connectivity have, in theory, higher resistance and resilience to environmental changes (Landi et al. 2018). Therefore, we can assume that the ecological networks of lowland lakes with higher connectance and connectivity are probably more stable and resilient to environmental changes than high‐altitude lakes. This hypothesis is of primary interest to lake managers who recognize that lakes are facing rapid changes (e.g., global warming, tourist overcrowding, water abstraction, and eutrophication).

### 4.3 Species Selection Is More Important in Lowland Than in High‐Altitude Lakes

Communities in high‐altitude lakes were less influenced by species selection than those in lowland lakes. This result is supported by the fact that 10% of lowland lakes' communities were shaped by species selection, compared to only 5% for high‐altitude lakes according to the approach of Stegen et al. (2013). This is further supported by the variance partitioning analysis showing that 50% of the variability in lowland communities was explained by local factors (chemical and hydro‐morphological parameters) compared to only 8% in high‐altitude lakes (Figure 6).

Strong environmental gradients generally lead to greater species selection (Lindström and Langenheder 2012; Nemergut et al. 2013), and lowland lakes can have stronger gradients than high‐altitude lakes. For example, if we consider lake surface area, that of lowland lakes varies by two orders of magnitude (from 1,450,000 m^2^ for Lake Bled to 580,000,000 m^2^ for Lake Geneva) and only one order of magnitude in high‐altitude (from 3806 m^2^ to 131,663 m^2^). This difference can lead to the presence of a greater variety of niches in large environments than in smaller ones, and therefore to more different species richness and community compositions in lowlands (Bolgovics et al. 2016). In addition, biotic factors *such as grazing of diatoms by invertebrates and fish should be* limited in high‐altitude compared to lowland lakes, and this is an additional factor that promotes more species selection in lowland than in high‐altitude. Conversely, if we consider nutrients as the level of total phosphorus in lowland and high‐altitude lakes, the two types of lake vary similarly by an order of magnitude in lowland (0.004 μgP/l in Lake Faaker to 0.233 μgP/l in Lake Geneva) and high‐altitude lakes (0.001 to 0.043 μgP/l). Another counter example is given with residence time, a decisive descriptor of lake functioning, which varies by two orders of magnitude (from 1.8 to 180 days) in high‐altitude lakes, whereas it varies by one order of magnitude in lowlands (620 days in Lake Mondsee to 9809 in Lake Garda), with conductivity (ranging from 9 to 1000 μs/cm in high‐altitude and from 174 to 352 μs/cm in lowlands). This tends to moderate this explanation for the smaller influence of species selection in high‐altitude.

## 5 Conclusions

Communities in high‐altitude lakes were mostly driven by stochastic processes, unlike those in lowland lakes, supporting the hypothesis on extreme conditions causing a severe community bottleneck in high‐altitude lakes. While dispersion was equally limiting in both lake types, homogenizing dispersal was more significant in lowland lakes, supporting the hypothesis of greater connectivity via anthropogenic and biotic vectors in lowland than in high‐altitude lakes. Finally, the communities' diversities, connectivity, and connectance of ecological networks in lowland lakes may make them more stable and resilient to environmental changes than high‐altitude lakes (Landi et al. 2018). However, we must acknowledge that the spatial distribution of the sampled lakes was heterogeneous, and this may influence our results.

These results have implications on the use of diatom communities for monitoring human impact in lakes. In high‐altitude lakes, where stochastic processes dominate, monitoring human activities (through, for instance, nutrient level) would be challenging. However, benthic diatoms remain a useful tool for monitoring lowland lakes and join the long‐standing tradition of diatom monitoring in lakes (Hoffman 1994; Marchetto et al. 2009).

Finally, the combined use of metrics estimating diversity, statistical analyses to identify ecological processes structuring communities, and inference of ecological networks provide valuable information on biological communities that, once disseminated to lake managers, will enable them to better understand the vulnerabilities of lakes, which are facing significant global changes.

## Author Contributions

**F. Rimet:** conceptualization (lead), data curation (lead), formal analysis (lead), funding acquisition (lead), investigation (lead), methodology (lead), project administration (lead), resources (lead), visualization (lead), writing – original draft (lead), writing – review and editing (equal). **C. Lemonnier:** writing – review and editing (supporting). **B. Alric:** formal analysis (supporting), investigation (supporting), writing – review and editing (supporting).

## Conflicts of Interest

The authors declare no conflicts of interest.

## Supporting information


**Data S1:** ece371977‐sup‐0001‐AppendixS1.zip.

## Data Availability

The data that support the findings of this study and Supporting Information are openly available at: https://doi.org/10.57745/0TVDYI.
