# Supplementary material for: Stochasticity Shapes Microbial Communities in High‐Altitude Lakes, Whereas Species Selection and Homogenization Dispersal Are More Important in Lowland Lakes: Case of Benthic Diatoms in Alpine Lakes
Source: Ecol Evol. 2025 Aug 27;15(9):e71977. doi: 10.1002/ece3.71977 (PMC12389866; doi:10.1002/ece3.71977)
Supplement: Supplementary file 1 — Data S1: ece371977‐sup‐0001‐AppendixS1.zip. [file ECE3-15-e71977-s001.zip › Supp data S4-ASVs accumulation curves.pdf]

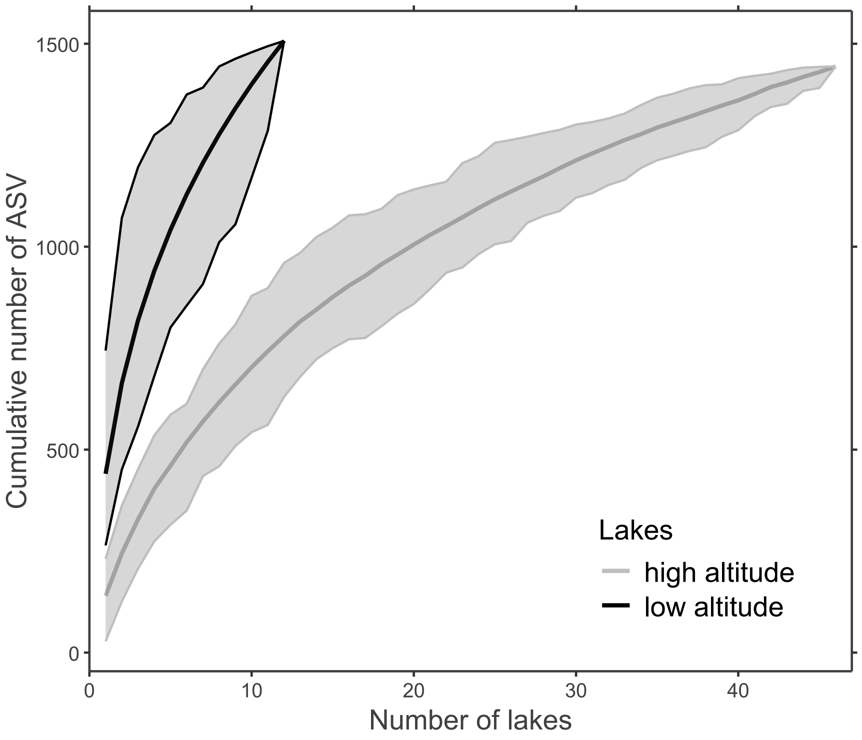

Supplementary data S5:

ASVs accumulation curves of high-altitude and lowland lakes. Solid line gives the average and the envelope gives the upper and lower values of the 100 random permutations. Observed richness is 1444 ASVs for high-altitude lakes and 1507 for lowland lakes. Chao2 is 2079 ASVs for high-altitude and 2273 for lowland lakes.
